# Blood pressure components and the risk for proteinuria in Japanese men: The Kansai Healthcare Study

**DOI:** 10.1016/j.je.2016.10.010

**Published:** 2017-07-11

**Authors:** Mikiko Shibata, Kyoko Kogawa Sato, Shinichiro Uehara, Hideo Koh, Shigeki Kinuhata, Keiko Oue, Hiroshi Kambe, Michio Morimoto, Tomoshige Hayashi

**Affiliations:** a Preventive Medicine and Environmental Health, Osaka City University Graduate School of Medicine, Osaka, Japan; b Hematology, Osaka City University Graduate School of Medicine, Osaka, Japan; c Medical Education and General Practice, Osaka City University Graduate School of Medicine, Osaka, Japan; d Kansai Health Administration Center, Nippon Telegraph and Telephone West Corporation, Osaka, Japan

**Keywords:** Blood pressure components, Proteinuria, Chronic kidney disease, Prospective cohort study, Epidemiology

## Abstract

**Background:**

We examined prospectively which of the four blood pressure (BP) components (systolic BP [SBP], diastolic BP [DBP], pulse pressure [PP], and mean arterial pressure [MAP]) was best in predicting the risk of proteinuria.

**Methods:**

This prospective study included 9341 non-diabetic Japanese middle-aged men who had no proteinuria and an estimated glomerular filtration rate ≥60 mL/min/1.73 m^2^ and were not taking antihypertensive medications at entry. Persistent proteinuria was defined if proteinuria was detected two or more times consecutively and persistently at the annual examination until the end of follow-up. We calculated the difference in values of Akaike's information criterion (ΔAIC) in comparison of the BP components-added model to the model without them in a Cox proportional hazards model.

**Results:**

During the 84,587 person-years follow-up period, we confirmed 151 cases of persistent proteinuria. In multiple-adjusted models that included a single BP component, the hazard ratios for persistent proteinuria for the highest quartile of SBP, PP, and MAP were 3.11 (95% confidence interval [CI], 1.79–5.39), 1.87 (95% CI, 1.18–2.94), and 2.21 (95% CI, 1.33–3.69) compared with the lowest quartile of SBP, PP, and MAP, respectively. The hazard ratio for the highest quartile of DBP was 2.69 (95% CI, 1.65–4.38) compared with the second quartile of DBP. Of all models that included a single BP component, those that included SBP alone or DBP alone had the highest values of ΔAIC (14.0 and 13.1, respectively) in predicting the risk of persistent proteinuria.

**Conclusions:**

Of all BP components, SBP and DBP were best in predicting the risk of persistent proteinuria in middle-aged Japanese men.

## Highlights

• We examined which blood pressure (BP) components increased risk of proteinuria.
• We used systolic BP, diastolic BP, pulse pressure, and mean arterial pressure.
• Systolic and diastolic BP were the best predictors of persistent proteinuria.

## Introduction

Hypertension is a well-established major risk factor for cardiovascular disease.^1^, ^2^ Although the chief criteria for defining hypertension have been based on systolic blood pressure (SBP) and diastolic blood pressure (DBP), it might be important for better prediction to consider two additional blood pressure (BP) components: a pulsatile component, such as pulse pressure (PP), and a steady component, such as mean arterial pressure (MAP).^3^ In epidemiological studies that have compared these BP components (SBP, DBP, PP, and MAP) individually as predictors of risk for cardiovascular diseases, SBP or PP has been reported to be associated with coronary heart disease,^4^, ^5^, ^6^, ^7^, ^8^, ^9^, ^10^, ^11^ whereas SBP has been associated with stroke.^4^, ^5^, ^6^, ^7^, ^8^, ^12^

Hypertension is also associated with the risk of chronic kidney disease,^13^ and a few prospective studies have reported the association between various BP components and the decline of glomerular filtration rate.^14^, ^15^, ^16^, ^17^ Although proteinuria is a component of chronic kidney disease and has been associated with the incidence of cardiovascular disease, end-stage renal disease, and all-cause mortality,^18^, ^19^, ^20^, ^21^, ^22^, ^23^ it is not known which of the four BP components is best in predicting the risk of incident proteinuria. Only two longitudinal studies have examined the associations between the four BP components and future albuminuria.^24^, ^25^ However, as both studies measured urinary albumin excretion only at the end point of their analytic cohorts, they have not examined the association between these BP components and incident risk of albuminuria. To our knowledge, no prospective cohort study has compared various BP components as predictors of risk for incident proteinuria.

We examined the relation of BP components (SBP, DBP, PP and MAP), both individually and combined, to the risk of the incidence of proteinuria in an 11-year prospective observational study in apparently healthy middle-aged Japanese men. To avoid the influence of subjects with transient proteinuria, we used the definition of persistent proteinuria, which was defined as proteinuria detected two or more times consecutively and persistently at the annual examination until the end of follow-up.

## Methods

### Study population

The study population was Japanese men enrolled in the Kansai Healthcare Study, which is an ongoing prospective cohort study examining risk factors for cardiometabolic diseases.^26^, ^27^ Between April 2000 and March 2001, 12,647 men aged 40–55 years who were employees of a company in the Kansai area of Japan and who were considered to be involved in sedentary jobs at entry were enrolled in this study. All employees of this company aged 40 years or older have undergone annual medical checkups because Japanese law requires it. When the data based on only the results of these annual health screenings are used, Japanese ethical guidelines for epidemiological research do not require written informed consent from participants. The protocol for this research was reviewed and approved by the Human Subjects Review Committee, Osaka City University.

For current analysis, we included 10,019 Japanese men who had no proteinuria, an estimated glomerular filtration rate (eGFR) ≥60 mL/min/1.73 m^2^, a fasting plasma glucose <126 mg/dL, and were not taking hypoglycemic medications, insulin, or antihypertensive medications. We excluded 320 men who did not have any medical checkups during the follow-up period and 358 men who had missing covariate information at baseline. Thus, the analytic cohort consisted of 9341 men.

### Data collection and measurements

The clinical examination consisted of medical history; physical examination; anthropometric measurements; self-reported questionnaires on lifestyle characteristics, such as smoking habit, alcohol consumption, and regular leisure-time physical activity; measurements of fasting plasma glucose and serum creatinine; and dipstick urinalysis. Trained nurses carried out all measurements.

BP was measured in a sitting position with an automatic sphygmomanometer (BP-203RV; Omron Colin, Tokyo, Japan, and Udex-super; ELK Osaka, Japan) after about 5 min of rest. The Spearman's correlation between the former device and a standard mercury sphygmomanometer was 0.985 for SBP and 0.976 for DBP. The Spearman's correlation between the latter device and a standard mercury-column sphygmomanometer was 0.997 for SBP and 0.976 for DBP. If the initial reading showed hypertension, BP was measured again, and the subject's BP was defined as the lower of the two readings after the second measurement. Hypertension was defined as SBP ≥140 mm Hg or DBP ≥90 mm Hg.^28^ PP was defined as SBP minus DBP. MAP was calculated as DBP plus one-third PP.

Urine samples were collected as clean-catch, mid-stream, and random urine specimens. The results of the dipstick urinalysis were interpreted as negative, ±, 1+, 2+, 3+, or 4+. Blood samples were obtained after a 12-h overnight fast. Serum creatinine was mainly measured by an enzymatic method using a Hitachi 7350 automatic chemistry analyzer (Hitachi Ltd., Tokyo, Japan). As serum creatinine was also measured by the Jaffe method in 1799 subjects at the baseline examination, the Jaffe values were recalibrated to correspond to enzymatic values using the following equation developed by the analytical laboratory: serum creatinine (mg/dL, enzymatic method) = 1.02 × serum creatinine (mg/dL, Jaffe method) – 0.25 (r = 0.9996). Then eGFR was calculated using the Modification of Diet in Renal Disease study equation for Japanese persons.^29^ Body mass index (BMI) was calculated as weight in kilograms divided by the square of the height in meters.

Self-reported questionnaires about lifestyle characteristics consisted of questions regarding smoking habit, drinking habit, and regular leisure-time physical activity. As for smoking habits, subjects were categorized as non-smokers, past smokers, or current smokers. The questions about drinking habit assessed the weekly frequency of alcohol consumption and the usual amount of alcohol consumed per drinking day according to Japanese standard drinks. One Japanese standard drink is 23 g of ethanol. Daily alcohol consumption was calculated as (the quantity consumed per drinking day) × (the weekly frequency of alcohol consumption)/7. As for regular leisure-time physical activity, the single-item questionnaire had three possible answers: rarely, sometimes, and regular (that is, at least once weekly). We classified subjects as engaging in leisure-time physical activity at least once weekly or less than once weekly. The validity of this questionnaire has been described in detail previously.^26^

### Outcome

Proteinuria on urine dipstick examination was defined as 1+ or higher (30 mg/dL or higher)^30^ at the annual medical check-up. We used the definition of persistent proteinuria to exclude transient proteinuria: “persistent proteinuria” was defined if proteinuria was detected two or more times consecutively and persistently at the annual examination until the end of follow-up.

### Statistical analysis

To assess the difference in baseline characteristics between subjects who developed persistent proteinuria and those who did not, we used the unpaired t-test, Mann–Whitney test, or chi-squared test (Table 1). Multivariate Cox proportional hazards models were used to investigate the association between BP components and the incidence of persistent proteinuria. Follow-up of each subject was continued until the diagnosis of the outcome occurrence or until the 11-year follow-up examination from April 1, 2011 to March 31, 2012, whichever came first. We evaluated nonlinear effects of continuous independent variables using fractional polynomials^31^ or by plotting the regression coefficients against the variables.^32^ In the Cox proportional hazards models of Table 2, BMI, alcohol consumption, DBP, and MAP did not fulfill the linearity assumption. As the association between BMI and risk of persistent proteinuria was a U-shaped association, the BMI level was classified into seven categories: <18.0, 18.0–19.9, 20.0–21.9, 22.0–23.9, 24.0–25.9, 26.0–27.9, and ≥28.0 kg/m^2^. Regarding drinking habits, except nondrinkers, subjects were classified into tertiles of daily alcohol consumption levels: 0.1–16.4, 16.5–42.7, and ≥42.8 g ethanol/day. Each BP component was categorized by quartile in all models of Table 2, Table 3. Therefore, we included the following variables into the models: single BP component or two BP components as categorical variables, age, BMI categories (<18.0, 18.0–19.9, 20.0–21.9, 22.0–23.9, 24.0–25.9, 26.0–27.9, and ≥28.0 kg/m^2^), fasting plasma glucose, smoking habits (non-smokers, past smokers, or current smokers), regular leisure-time activity (yes or no), drinking habits (non-drinkers, 0.1–16.4, 16.5–42.7, and ≥42.8 g ethanol/day), and eGFR at baseline. The proportional hazards assumption was confirmed by the insertion of time-dependent covariates or by the Schoenfeld residuals plot and Schoenfeld residuals test.^33^ The presence of an effect-modification was tested by the insertion of first-order interaction terms into appropriate proportional hazards models. We examined the significance of the interaction terms of SBP × DBP and PP × MAP in the models including these two BP components (Table 2). These interaction terms did not improve model fit. Multicollinearity was assessed using the variance inflation factor.^34^ There was no evidence of multicollinearity. We checked for outliers by plotting the likelihood displacement values and LMAX values of all independent variables.^33^ The linear trends in risks of quartiles of BP components were evaluated by entering the median value for each quartile category as continuous variables.

**Table 1:** Baseline characteristics of study participants according to whether or not persistent proteinuria developed during the follow-up period.

	Total	persistent proteinuria^a^		*P* value
		(−)	(+)	
Number	9341	9190	151	
Age, years	48.2 (4.2)	48.2 (4.2)	48.8 (4.0)	0.074
Body mass index, kg/m^2^	23.2 (2.8)	23.2 (2.8)	24.4 (3.2)	<0.001
Systolic blood pressure, mm Hg	127.6 (17.7)	127.5 (17.6)	135.5 (19.1)	<0.001
Diastolic blood pressure, mm Hg	79.7 (11.8)	79.6 (11.7)	84.0 (14.7)	<0.001
Pulse pressure, mm Hg	47.9 (12.5)	47.9 (12.4)	51.4 (14.3)	<0.001
Mean arterial pressure, mm Hg	95.6 (12.7)	95.5 (12.7)	101.2 (14.8)	<0.001
Hypertension, %^b^	28.1	27.8	48.3	<0.001
Fasting plasma glucose, mg/dL	97.2 (9.2)	97.2 (9.2)	99.5 (9.1)	0.002
Serum creatinine, mg/dL	0.79 (0.11)	0.79 (0.11)	0.77 (0.12)	0.015
Estimated glomerular filtration rate, mL/min/1.73 m^2^	84.8 (14.1)	84.8 (14.0)	87.7 (16.4)	0.011
Daily alcohol consumption, g ethanol/day^c^	23.0 (3.3–46.0)	23.0 (3.3–46.0)	24.6 (8.2–46.0)	0.214
Drinking habit, %	85.0	85.1	84.1	0.744
Regular leisure-time physical activity, %	17.9	18.0	9.3	0.005
Smoking habits,%				
Nonsmokers	21.2	21.2	19.2	
Past smokers	21.6	21.7	17.2	0.254
Current smokers	57.2	57.1	63.6	

Data are presented as mean (standard deviation), median (25th to 75th percentiles), or % and compared using unpaired t-test, Mann–Whitney test, or chi-squared test.

a Persistent proteinuria was defined as proteinuria detected two or more times consecutively and persistently at the annual examination until the end of follow-up.

b Hypertension was defined as systolic blood pressure ≥140 mm Hg or diastolic blood pressure ≥90 mm Hg.

c Daily alcohol consumption was calculated as [(the quantity consumed per drinking day) × (the weekly frequency of alcohol consumption)/7].

**Table 2:** Comparison of four blood components in predicting the incidence of persistent proteinuria^a^.

Model	Incidence rate^b^ (case/person-years)	Multiple-adjusted hazard ratio (95% CI)^c^	*P* value
Base model^d^ + single BP component			
SBP, mm Hg			
Quartile 1 (–115)	0.83 (18/21617)	1.00 (reference)	
Quartile 2 (116–126)	1.56 (35/22416)	1.85 (1.04–3.28)	0.036
Quartile 3 (127–138)	1.63 (35/21501)	1.77 (0.99–3.17)	0.055
Quartile 4 (139–)	3.31 (63/19053)	3.11 (1.79–5.39)	<0.001
P for trend		<0.001	
DBP, mm Hg			
Quartile 1 (–71)	1.52 (33/21689)	1.65 (0.97–2.83)	0.067
Quartile 2 (72–80)	1.00 (23/23027)	1.00 (reference)	
Quartile 3 (81–87)	1.57 (32/20441)	1.44 (0.84–2.47)	0.184
Quartile 4 (88–)	3.24 (63/19430)	2.69 (1.65–4.38)	<0.001
P for trend		0.005	
PP, mm Hg			
Quartile 1 (–40)	1.25 (30/24091)	1.00 (reference)	
Quartile 2 (41–47)	1.57 (33/21061)	1.23 (0.75–2.03)	0.407
Quartile 3 (48–55)	1.75 (35/20049)	1.32 (0.81–2.16)	0.270
Quartile 4 (56–)	2.73 (53/19386)	1.87 (1.18–2.94)	0.007
P for trend		0.005	
MAP, mm Hg			
Quartile 1 (–86.7)	1.05 (23/21972)	1.00 (reference)	
Quartile 2 (86.8–95.0)	1.49 (32/21437)	1.30 (0.76–2.24)	0.342
Quartile 3 (95.1–103.3)	1.58 (33/20884)	1.31 (0.75–2.27)	0.339
Quartile 4 (103.4–)	3.10 (63/20294)	2.21 (1.33–3.69)	0.002
P for trend		0.001	
Base model^d^ + two BP components			
SBP and DBP			
SBP, mm Hg			
Quartile 1 (–115)		1.00 (reference)	
Quartile 2 (116–126)		2.23 (1.22–4.07)	0.009
Quartile 3 (127–138)		2.07 (1.07–3.97)	0.030
Quartile 4 (139–)		3.19 (1.61–6.30)	0.001
P for trend		0.002	
DBP, mm Hg			
Quartile 1 (–71)		2.14 (1.23–3.74)	0.007
Quartile 2 (72–80)		1.00 (reference)	
Quartile 3 (81–87)		1.24 (0.71–2.15)	0.446
Quartile 4 (88–)		1.94 (1.12–3.34)	0.017
P for trend		0.703	
PP and MAP			
PP, mm Hg			
Quartile 1 (–40)		1.00 (reference)	
Quartile 2 (41–47)		1.19 (0.72–1.96)	0.493
Quartile 3 (48–55)		1.21 (0.73–1.99)	0.457
Quartile 4 (56–)		1.54 (0.96–2.49)	0.075
P for trend		0.067	
MAP, mm Hg			
Quartile 1 (–86.7)		1.00 (reference)	
Quartile 2 (86.8–95.0)		1.27 (0.74–2.19)	0.390
Quartile 3 (95.1–103.3)		1.23 (0.71–2.14)	0.462
Quartile 4 (103.4–)		1.94 (1.14–3.29)	0.015
P for trend		0.010	

BP, blood pressure; CI, confidence interval; DBP, diastolic blood pressure; MAP, mean arterial pressure; PP, pulse pressure; SBP, systolic blood pressure.

a Persistent proteinuria was defined if proteinuria was detected two or more times consecutively and persistently at the annual examination until the end of follow-up.

b Incidence rates are expressed as the incidence per 1000 person-years.

c Adjusted for base model + single BP component or two BP components.

d Base model included age, body mass index categories (<18.0, 18.0–19.9, 20.0–21.9, 22.0–23.9, 24.0–25.9, 26.0–27.9, ≥28.0), fasting plasma glucose, smoking habits (non-smokers, past smokers, current smokers), regular leisure-time activity (yes or no), drinking habits (non-drinkers, 0.1–16.4, 16.5–42.7, ≥42.8 g ethanol/day), and estimated glomerular filtration rate at baseline.

**Table 3:** The effects of adding each BP component to base model in predicting the incidence of persistent proteinuria^a^.

Model 1	Model 2	ΔAIC
Base model	Base model + SBP	14.0
Base model	Base model + DBP	13.1
Base model	Base model + PP	2.0
Base model	Base model + MAP	6.4
Base model	Base model + SBP + DBP	20.0
Base model	Base model + PP + MAP	3.8

AIC, Akaike's information criterion; BP, blood pressure; DBP, diastolic blood pressure; MAP, mean arterial pressure; PP, pulse pressure; SBP, systolic blood pressure.

We calculated ΔAIC for persistent proteinuria by comparing model 2 to model 1. ΔAIC was used to evaluate which BP components or combined BP components, SBP, DBP, PP, MAP, SBP + DBP or PP + MAP, were superior as the risk for incident persistent proteinuria. Higher value of ΔAIC indicated better model fit.

Base model included age, body mass index categories (<18.0, 18.0–19.9, 20.0–21.9, 22.0–23.9, 24.0–25.9, 26.0–27.9, ≥28.0), fasting plasma glucose, smoking habits (non-smokers, past smokers, current smokers), regular leisure-time activity (yes or no), drinking habits (non-drinkers, 0.1–16.4, 16.5–42.7, ≥42.8 g ethanol/day), and estimated glomerular filtration rate at baseline, but no BP component. Model 2 included each BP component quartile in addition to all variables of base model.

a Persistent proteinuria was defined if proteinuria was detected two or more times consecutively and persistently at the annual examination until the end of follow-up.

We examined which BP component had a strong association with the incidence of persistent proteinuria. Difference in values of Akaike's information criterion (ΔAIC) was used to measure the improvement of goodness of fit in comparison of BP components-added models to base model without BP component because AIC can be used to compare non-nested models (Table 3).^35^ An absolute ΔAIC value more than 2.5 indicates a meaningful difference of goodness of fit between the models.^35^ Higher value of ΔAIC indicates better model fit.

We calculated the 95% confidence interval for each hazard ratio. All *P* values were two-tailed. Statistical analyses were performed using Stata MP, Version 13.1 (Stata Corp., College Station, TX, USA).

## Results

Table 1 reports the baseline characteristics of the subjects by incident persistent proteinuria status. Subjects who developed persistent proteinuria had higher mean values of SBP, DBP, PP, and MAP than those who did not. The group of subjects who developed persistent proteinuria had a higher proportion of hypertension and higher mean BMI and eGFR at baseline.

During the 84,587 person-years of follow-up period, 151 subjects had persistent proteinuria. The incidence rate and multiple-adjusted hazard ratios are shown in Table 2. In multiple-adjusted models including a single BP component, SBP, DBP, PP, or MAP, higher levels of SBP, PP, and MAP were associated with an increased risk of persistent proteinuria, and the association between DBP and risk of persistent proteinuria was a U-shaped association. When SBP and DBP were included simultaneously in the model, both SBP and DBP were independently associated with an increased risk of persistent proteinuria. Higher levels of SBP were associated with an increased risk of persistent proteinuria, and the association between DBP and risk of persistent proteinuria was a U-shaped association. When PP and MAP were included simultaneously in the model, both PP and MAP were associated with an increased risk of persistent proteinuria, but the association between PP and risk of persistent proteinuria did not reach statistical significance.

We examined which BP component or combined BP component (i.e., SBP, DBP, PP, MAP, SBP + DBP, or PP + MAP) was the best predictor of incident persistent proteinuria. ΔAIC was calculated as the AIC of the model that did not include any BP components minus the AIC of the model that included any one BP component or combined BP component (Table 3). Of all the models that included a single BP component, those that included SBP alone or DBP alone showed the best improvement of goodness of fit for predicting incident persistent proteinuria (ΔAIC 14.0 and 13.1, respectively). On the other hand, PP alone did not show improvement of goodness of fit for predicting incident persistent proteinuria. When we examined which single BP component or combined BP component was the best predictor of incident persistent proteinuria, the model that included SBP + DBP showed the best goodness of fit to predict the incident persistent proteinuria. The model that included PP + MAP was not superior to models that included SBP alone or DBP alone.

## Discussion

These prospective data demonstrated that all the BP components (SBP, DBP, PP, and MAP) were associated with an increased risk of persistent proteinuria. Higher levels of SBP, PP, and MAP were associated with an increased risk of persistent proteinuria. On the other hand, the association between DBP and persistent proteinuria had a U-shaped association. Of all models that included the single BP component, SBP and DBP were best in predicting the risk of persistent proteinuria. Of all models that included the single BP component or combined BP component, the model that included SBP + DBP showed the best goodness of fit to predict the risk of the incident persistent proteinuria. These associations were independent of age, BMI, fasting plasma glucose level, smoking habits, regular leisure-time activity, drinking habits, and eGFR level.

Only two longitudinal studies have shown the relationship between the four BP components and future albuminuria.^24^, ^25^ Farasat et al^24^ reported in the Baltimore Longitudinal Study of Aging that PP was the strongest predictor of 24-h urinary albumin excretion in men, and that each BP component was not associated with urinary albumin excretion in women. Although they have used serial BP measurements during 1–22 years (median, 5 years) preceding urinary albumin excretion measurement as an independent variable, they measured urinary albumin excretion only at the end-point of their analytic cohort. Subjects in their study were older than our subjects, and PP becomes increasingly greater with advancing age,^36^, ^37^ which may explain the difference between their results and ours. Tsakiris et al^25^ showed in the Three Areas Study in Greece that SBP, DBP, and MAP at entry were associated with microalbuminuria measured at the end of the 12-year follow-up period. They did not measure urinary albumin excretion at entry, and the follow-up rate was low (57%). As both studies did not assess the presence or absence of albuminuria at entry, they have not examined the relationship between BP components and the incident risk of albuminuria.

One of the strengths of this study was that we examined the association between BP components and incident persistent proteinuria to avoid the influence of subjects with transient proteinuria. Previous studies^24^, ^25^ on the association between BP components and the risk of albuminuria have used single measurements of urinary albumin excretion.

In this study, we did not identify why SBP and DBP were the best predictors of incident proteinuria. In general, chronic hypertension causes impairment of the renal autoregulation mechanism, which keeps renal blood flow and the glomerular filtration rate constant when blood pressure level increases.^38^ This impairment leads to glomerular hypertension and an increase in proteinuria.^38^ Although SBP rises steadily with age, DBP rises until around age 50–60 years and thereafter falls progressively. PP becomes increasingly greater with advancing age.^36^, ^37^ In our study, subjects were 40–55 years old at entry, which may explain why PP was not a useful predictor.

Some cohort studies have reported that PP is a stronger predictor of coronary heart disease in older people,^9^, ^10^, ^11^ but others have shown that PP has less predictive utility than the other BP components and that SBP is a more useful predictor.^4^, ^5^, ^6^, ^7^, ^8^ As for stroke, some previous cohort studies have reported that SBP is a stronger predictor than PP.^4^, ^5^, ^6^, ^7^, ^8^, ^12^ Regarding chronic kidney disease, only a few cohort studies that have related BP components to renal function decline are available.^14^, ^15^, ^16^, ^17^ Two cohort studies that focused on only elderly subjects have reported that SBP was the strongest predictor of deterioration in kidney function.^14^, ^17^ Our previous study in the same cohort study reported that in subjects aged 40–55 years at baseline, DBP and MAP were the most useful predictors for incidence of an eGFR of <60 mL/min/1.73 m^2^.^16^ In our current study, the relationship of BP components to risk for proteinuria was different from that for the decline of eGFR. These results suggest that each BP component might have different roles in renal damage, in damage to other organs, or at different ages.

Several limitations should be noted. First, because all of the subjects were middle-aged Japanese men working at the same company, our results may not be applied to women, older men, or other ethnic groups. In addition, our results may underestimate the real relationship between BP components and proteinuria risk because of the healthy worker effect.^39^ Second, because our study used a single office BP at baseline, we could not adjust home BP nor 24-h BP measured using ambulatory BP monitoring and changes in such BP components. Third, as we measured proteinuria using a dipstick instead of a quantitative and/or semi-quantitative method to detect albuminuria, the subjects with incident proteinuria may include a number of those with false-positive and false-negative proteinuria. However, qualitative dipstick testing is convenient, easy to perform, and commonplace in clinical practice and large epidemiological studies. In addition, we evaluated persistent proteinuria to reduce the overestimation of false-positive proteinuria as much as possible.

In summary, of all models that included a single BP component, SBP and DBP were the best predictors for persistent proteinuria. Of all models that included a single BP or combined BP components, the model that included SBP + DBP was the best predictor for persistent proteinuria in middle-aged Japanese men. Further study will be needed to examine this association in other ethnic groups and in older age groups.

## Conflicts of interest

None declared.
